# Supplementary material for: From sequence to enzyme mechanism using multi-label machine learning
Source: BMC Bioinformatics. 2014 May 19;15:150. doi: 10.1186/1471-2105-15-150 (PMC4229970; doi:10.1186/1471-2105-15-150)
Supplement: Additional file 2 — Java code of ml2db. Additional file ml2db_code.tar.gz contains the Java source code to run the multi-label machine learning experiments and save the results to database. The code’s Javadoc is included. [file 1471-2105-15-150-S2.zip › additional file 2/ml2db/utils/doc/uk/ac/standrews/utils/main/collectionutils/setutils/class-use/Set.html]

Uses of Class uk.ac.standrews.utils.main.collectionutils.setutils.Set


JavaScript is disabled on your browser.


- Overview
- Package
- Class
- Use
- Tree
- Deprecated
- Index
- Help

- Prev
- Next

- Frames
- No Frames

- All Classes

## Uses of Class uk.ac.standrews.utils.main.collectionutils.setutils.Set

- Packages that use Set

  | Package | Description |
  | --- | --- |
  |  |  |
  | --- | --- |
  | uk.ac.standrews.utils.main.collectionutils.setutils |  |
  | uk.ac.standrews.utils.test.setutils |  |
- - ### Uses of Set in uk.ac.standrews.utils.main.collectionutils.setutils

    Methods in uk.ac.standrews.utils.main.collectionutils.setutils that return Set

    | Modifier and Type | Method and Description |
    | --- | --- |
    |  |  |
    | --- | --- |
    | `Set` | Set.`clone()` |

    Methods in uk.ac.standrews.utils.main.collectionutils.setutils that return types with arguments of type Set

    | Modifier and Type | Method and Description |
    | --- | --- |
    |  |  |
    | --- | --- |
    | `java.util.TreeSet<Set>` | SupersetsManager.`createSupersets()` |
    | `java.util.TreeSet<Set>` | SupersetsManager.`getSets()` |
    | `java.util.TreeSet<Set>` | Set.`getSubsets()` |
    | `java.util.TreeSet<Set>` | SupersetsManager.`getSupersets()` |

    Methods in uk.ac.standrews.utils.main.collectionutils.setutils with parameters of type Set

    | Modifier and Type | Method and Description |
    | --- | --- |
    |  |  |
    | --- | --- |
    | `void` | Set.`addSubset(Set newsetToAdd)` |
    | `int` | Set.`compareTo(Set otherSet)` |
    | `boolean` | Set.`contains(Set set)` |

    Constructor parameters in uk.ac.standrews.utils.main.collectionutils.setutils with type arguments of type Set

    Constructor and Description

    `Set(java.lang.String id,
    java.util.TreeSet<java.lang.String> elements,
    SupersetsManager manager,
    java.util.TreeSet<Set> subsets)`
  - ### Uses of Set in uk.ac.standrews.utils.test.setutils

    Methods in uk.ac.standrews.utils.test.setutils that return Set

    | Modifier and Type | Method and Description |
    | --- | --- |
    |  |  |
    | --- | --- |
    | `static Set` | SetTest.`getSet(java.lang.String id, java.util.TreeSet<java.lang.String> elements)` |
    | `static Set` | SetTest.`getSetA()` |
    | `static Set` | SetTest.`getSetAB()` |
    | `static Set` | SetTest.`getSetABC()` |
    | `static Set` | SetTest.`getSetAC()` |
    | `static Set` | SetTest.`getSetAD()` |

- Overview
- Package
- Class
- Use
- Tree
- Deprecated
- Index
- Help

- Prev
- Next

- Frames
- No Frames

- All Classes
